# Emotion Expression in Breast Cancer Support Seeking: Empirical Study of an Online Community

**DOI:** 10.2196/83674

**Published:** 2026-04-13

**Authors:** Anqi Xu, Ahmad Aljanaideh, Jennifer Xu, Haijing Hao

**Affiliations:** 1 Department of Computer Information Systems Bentley University Waltham, MA United States

**Keywords:** breast cancer, online cancer community, emotion, patient engagement, machine learning

## Abstract

**Background:**

Breast cancer affects millions of women and presents not only medical challenges but also emotional, financial, and social burdens. Beyond clinical treatment, patients increasingly turn to online cancer communities (OCCs) for informational support, emotional support, and shared coping strategies. OCCs help patients manage daily life and reduce psychological distress through shared experiences and empathetic engagement. Within these communities, emotional expressions serve as critical cues through which patients communicate their situations and needs with other OCC members.

**Objective:**

This study explores the relationship between emotions expressed in patients’ initial posts and community reactions and engagement in OCCs. Based on the emotions as social information (EASI) model and Plutchik’s Wheel of Emotions, this study investigates whether different emotions function as distinct social signals that lead to different patterns of member response, and whether emotions defined as opposites elicit opposing or similar engagement behaviors.

**Methods:**

This study examines how the 8 primary emotions expressed in an initial post—surprise, anticipation, joy, sadness, trust, disgust, fear, and anger—distinctively influence responses of members in OCCs. We collected data from a breast cancer community from 2002 to 2017 and analyzed 23,633 threads from 9137 patients with breast cancer. Using BERT (Bidirectional Encoder Representations From Transformers), we extract emotion scores from patients’ initial posts, measure community engagement across 5 response dimensions, and empirically analyze how different emotions are associated with user response behaviors.

**Results:**

The analysis shows that certain emotions, such as joy and anticipation, consistently elicit significant effects across all measured response categories (*P*<.001). Most other emotions, except disgust, also demonstrate significant effects in most categories (3 or 4 of 5 categories; *P*<.05). Consistent with the EASI perspective, different emotional expressions influence how community members allocate attention, effort, and timing in their responses in distinct ways. Moreover, for most pairs of opposite emotions (eg, surprise vs anticipation, joy vs sadness, and anger vs fear), the impacts on the 5 types of community responses move in parallel rather than opposite directions.

**Conclusions:**

By integrating Plutchik’s emotion framework with the EASI model, this study advances understanding of how emotional expressions function as social information and signals in OCCs. The findings show that emotions expressed in patients’ initial posts influence community engagement not only through emotional valence, but also through the situational context that community members can infer from the initial posts. In addition, modeling emotions as continuous intensity scales helps reveal how emotional strength amplifies engagement across multiple dimensions, including participation, effort, timing, and topical relevance. These insights extend prior OCC research beyond polarity-based sentiment analysis and offer practical implications for patients, community platforms, and health care practitioners seeking to better support peer interaction and psychosocial care in digital health environments.

## Introduction

Cancer is a life-threatening medical condition that can overwhelm patients with a wide range of challenges, including physical symptoms, emotional distress, financial difficulties, social isolation, etc [1-3]. For example, breast cancer, one of the most diagnosed cancers in women [4,5], traumatizes, threatens, debilitates, and devastates many female patients all over the world. Men could also endure breast cancer, with a likelihood or frequency much lower than that for women [6]. According to Breastcancer.org [7], about 32% of all new cancer cases each year are expected to be breast cancer, and it is estimated that 13% of American women will develop invasive breast cancer at some point in their lives. Although breast cancer typically has higher survival and recovery rates than some other types of cancer (eg, lung cancer and liver cancer), it usually requires ongoing care over an extended period of time [8].

The complexity of cancer care and the need for comprehensive support motivate society to provide patients with various forms of assistance in addition to medical treatments and care from physicians. Online cancer communities (OCCs) play a vital role in providing patients with cancer with technology-powered solutions to information sharing and social support [9-11]. In these communities, patients engage in interactions with other members through posting messages to share and obtain timely information (eg, treatments, symptoms, and coping strategies), medical or nonmedical experiences, care and empathy, encouragement, and companionship [10,12-18]. The tips they share about managing daily life with cancer, ranging from strategies for dealing with side effects to general nutrition advice, can help improve patients’ quality of life. In this sense, engaging in OCCs can contribute to better physical health outcomes and treatment adherence.

Previous research has explored online community engagement from multiple perspectives, such as active vs passive participation, churn behavior, life span in the community, the role of moderators in improving engagement, and community members’ responses to different types of initial posts [19-25]. In particular, it has been shown that other community members’ responses to initial posts are important to both the initial posters and the community as a whole, because these responses not only improve the initial posters’ satisfaction [26] and empowerment [27] but also contribute new information resources that help others [28,29]. Prior research on community members’ responses has examined the initial poster’s characteristics [21] and content [23,30], various types of social support expressed in the post content [19-22], and how participation can improve community members’ emotional states [10,16,31,32]. The emotional support and sense of belonging that patients receive and develop through interacting with other members in OCCs can reduce patients’ feelings of isolation and anxiety. These supports can also potentially lower the risk of depression among patients with cancer, which is crucial to their health and recovery [33-36]. Research has shown that emotions can affect a patient’s acceptance of the prescribed treatments or therapeutic plan, and influence their responses to the associated stresses and anxieties [37].

Importantly, emotional expressions in OCCs are not merely reflections of patients’ internal states but play a central role in affecting social interaction. Prior research conceptualizes emotional expressions as social information that helps others interpret ambiguous situations and decide whether and how to respond. In particular, the emotions as social information (EASI) model states that emotional expressions convey situational appraisals by signaling how individuals evaluate their situations, which allows observers to interpret ambiguous contexts and adjust their responses through inferential and affective processes [38]. Building on this view, Van Kleef et al [39] further argue that emotional expressions function as social signals that guide and coordinate others’ behavioral responses in social interaction, with different emotions signaling distinct behavioral responses. These perspectives are especially relevant in digitally mediated health communities, where contextual cues are limited and emotional expressions often serve as primary signals of patients’ needs, concerns, and circumstances.

However, most research on emotions in online communities polarized sentiments as being either positive or negative [31,40-45]. Such a dichotomy oversimplifies the complexity of human emotions, especially in situations where one must deal with highly stressful conditions such as cancer. Indeed, emotions can have multiple dimensions and are not always definitively positive or negative. For example, anger is commonly considered a negative emotion; yet, some studies have discovered its positive aspects [46]. Cancer can also evoke fear and sadness in patients when first diagnosed with cancer, and patients may later feel joy and relief when their conditions improve due to treatments. Patient emotions can also change, and their emotional intensity can vary over time [47,48], ranging from mild to strong. Patients experience these ups and downs and various emotions with changing intensities throughout their cancer management journey, and many choose to share their feelings on OCCs. It is important to recognize such complexity in both emotion types and emotion intensities expressed in patient posts and to examine how community members respond to posts that carry emotions.

**Figure 1:**
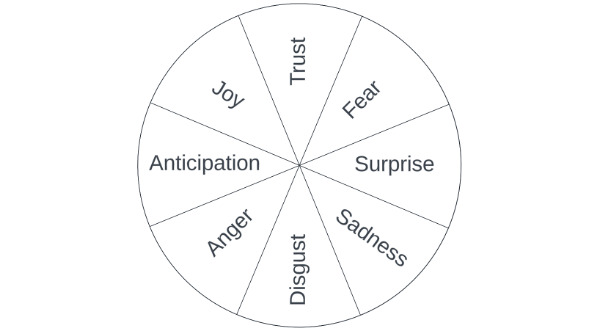
Plutchik’s 8 primary emotions.

To capture emotional complexity, we use Plutchik’s Wheel of Emotions [49,50] to examine a more comprehensive representation of patient emotions in the context of OCCs. This framework has been widely applied to analyze emotional expressions in online text [51-53]. Figure 1 presents Plutchik’s Wheel of Emotions, which consists of 8 distinct emotions: surprise, anticipation, joy, sadness, trust, disgust, fear, and anger [49]. In terms of community members’ responses, according to the EASI model and the derived social signal perspective, emotional expressions function as social information and social signals in online interaction, and different types of emotions may have varying impacts on how community members interpret and respond to patient posts. Drawing on Plutchik’s identification of distinct primary emotions and the EASI perspective, and taking emotion intensity into consideration, this study asks the following research question: RQ1: in OCC settings, are the type and intensity of emotions expressed in patients’ initial posts associated with different patterns of community engagement and response?

Plutchik arranges the emotions in a circular format, with each primary emotion having an opposite emotion directly across from it on the wheel, forming 4 pairs of opposing emotions [49,50]. We modify the original Plutchik’s Wheel of Emotions [49] and Plutchik’s Primary Emotion Dyads [54] to show the 8 primary emotions and their opposing counterparts. This study only focuses on the 8 primary emotions in Plutchik’s theory and does not consider other emotions, such as the secondary emotions that are the combinations of the primary emotions, as they are not the focus of this study. This structure reflects Plutchik’s conceptualization of emotions as adaptive reactions to 4 fundamental life problems: territoriality, temporality, identity, and hierarchy [48,55]. Each life problem can be evaluated as either an opportunity or a threat, and these alternative evaluations activate different adaptive functions and corresponding emotional reactions. Emotions thus reflect not only affective states but also action readiness, such as acceptance vs rejection or protection vs confrontation. Accordingly, territoriality may evoke surprise or anticipation; temporality may evoke sadness or joy; hierarchy may evoke fear or anger, and identity may evoke disgust or trust [49].

Within the cancer context, the territoriality problem refers to patients’ attempts to explore their uncertain health states. As cancer outcomes are often unpredictable, patients may experience anticipation while awaiting scan results or surprise when confronted with unexpected findings. Temporality-related situations reflect concerns about the limited duration of life, which activate patients’ emotions related to life longevity (joy) or loss (sadness) as the illness and treatment progress over time. The identity problem concerns inclusion and exclusion (eg, who or what is accepted or rejected), which may reflect trust or disgust toward one’s diagnosis, treatment options, or the opinions and support of others. Finally, hierarchy-related threats, such as the perceived power imbalance between oneself and an uncontrollable disease or condition, may elicit patients’ fear-oriented avoidance or anger-driven confrontation.

Plutchik’s Wheel of Emotions suggests that the same underlying circumstances can lead to opposing emotions through alternative adaptive appraisals. Hence, the same cancer-related situation can elicit opposite emotions, depending on how patients cognitively evaluate the situation and the adaptive reactions that are activated. In contrast, existing EASI-based research has mostly examined individual emotions separately (eg, different emotions signal distinct behavioral responses from observers) [39] and has not explicitly considered how pairs of opposite emotions operate in social interaction, especially in contexts where opposite emotions may be triggered by the same or highly similar situations. If the same situation can produce different emotional expressions, it is unclear whether observers interpret these emotions as signaling different situational contexts, which leads to different responses, or as alternative emotional reactions to the same underlying situation, which leads to similar responses. Although prior studies have linked individual emotions or polarized sentiments to engagement [31,40-45], they have not examined opposing emotions as social signals in breast cancer online communities. As a result, it remains theoretically unresolved whether opposite emotions function as equivalent or opposing social signals in affecting community members’ engagement behaviors, such as whether to respond, how quickly to respond, how much effort to invest, and how closely responses align with patients’ expressed concerns. To directly address this unresolved issue, we ask the following research question: RQ2: Do opposite emotions elicit opposing or similar engagement behaviors and responses from community members?

By addressing these 2 research questions, we seek to ascertain whether community members’ responses are driven primarily by the specific emotion expressed (ie, treating opposite emotions as distinct social signals) or by the underlying situational context inferred from the post (ie, treating opposite emotions as alternative expressions of the same situation).

In summary, this study is aimed at providing a comprehensive and fine-grained understanding of how different types of emotions and their intensities expressed in patients with cancer’s initial posts stimulate community responses in OCCs. By examining 8 primary emotions, this study moves beyond polarity-based sentiment analysis and uncovers the emotional mechanisms that affect patient engagement, peer support, and interaction dynamics in OCCs.

## Methods

### Study Design

**Figure 2:**
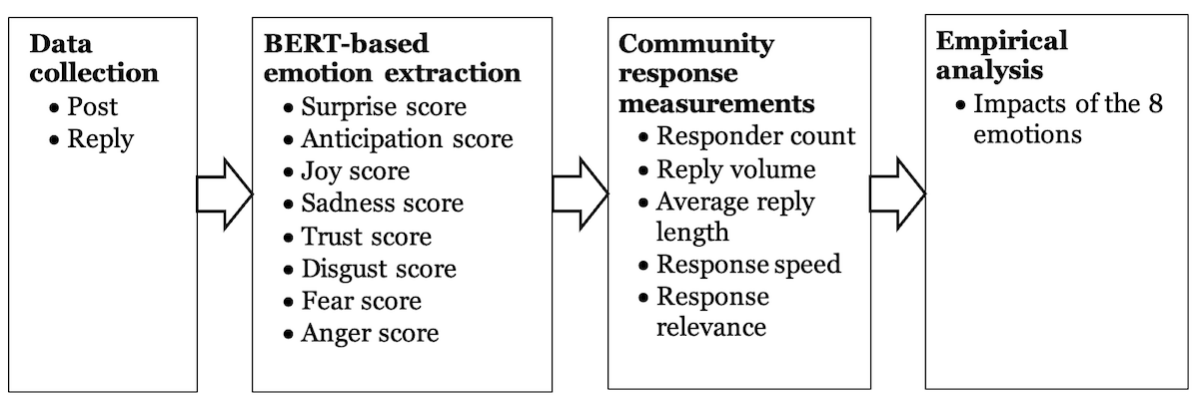
The overall approach. BERT: Bidirectional Encoder Representations From Transformers.

Figure 2 presents the overall process of our study, which includes 4 stages: collecting posts and replies from an OCC (a breast cancer community), extracting emotion scores from initial posts, gauging engagement behaviors by analyzing community responses, and empirically analyzing the effects of different emotions on user response behavior.

### Ethical Considerations

Our study uses publicly available data from an online breast cancer community, community.breastcancer.org. The research protocol was reviewed by the Institutional Review Board at Bentley University and was determined to be exempt from further review under the US Department of Health and Human Services regulations for the protection of human research participants (45 CFR 46.102[e][2][ii]), which applies to research involving observation of public behavior where the information recorded does not allow identification of the individuals.

As this study analyzed publicly accessible user-generated content and did not involve direct interaction with participants, informed consent was not required. To protect participant privacy, all data were deidentified before analysis. Usernames and other potentially identifiable information were removed, and findings are reported only in aggregated form to prevent identification of individual users.

Participants did not receive compensation because this study relied solely on publicly available online posts and did not involve recruitment or intervention.

### Data Collection

**Figure 3:**
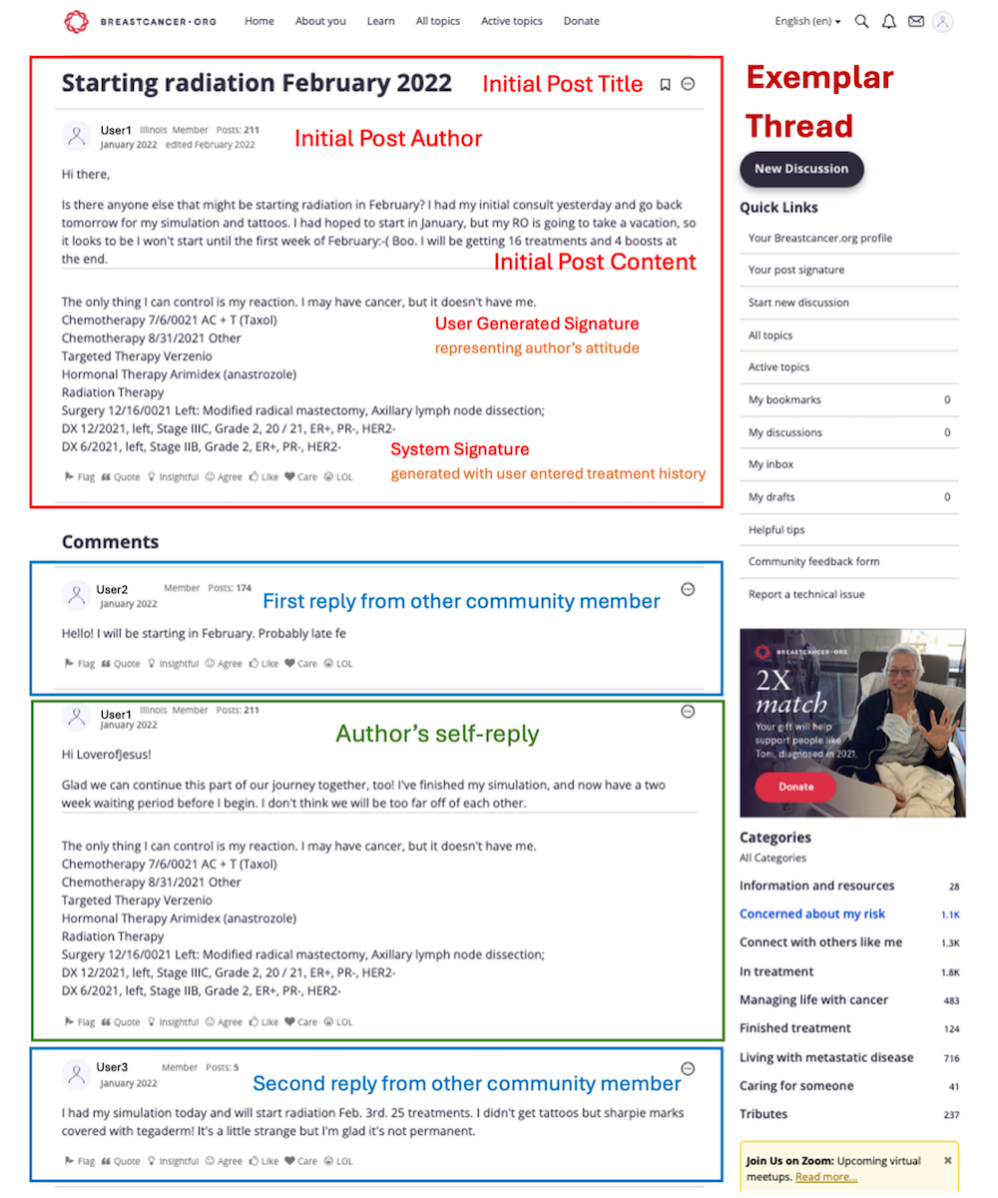
Exemplar thread from an online breast cancer community.

community.breastcancer.org is a prominent OCC for patients with breast cancer and covers a range of topics relevant to breast cancer, and provides a structured platform for patients to interact and exchange information. In this OCC, a patient in need of help typically starts a conversation (a.k.a., thread) by posting a message (a.k.a., initial post). Other members in the community can respond by adding their replies to the same thread. The initial poster may also post self-replies. Following each message (eg, the initial post and the replies) are the authors’ signatures. There are 2 types of signatures—user-generated signatures and system signatures. User-generated signatures are customized texts that members choose to display, while system signatures are automatically generated using the member’s self-reported diagnosis and treatment history. Figure 3 depicts a thread in community.breastcancer.org.

The forum consists of numerous threads, with each thread comprising an initial post followed by a chronological sequence of replies to that post. One patient may initiate multiple initial posts, and each initial post leads to 1 thread. Our dataset spans from October 21, 2002, to November 19, 2017, containing 101,171 threads initiated by 25,481 patients, with a total of 1,949,894 replies by the time of data collection. For the purposes of this study, we excluded threads with no replies and those whose initial posts were predominantly informational with minimal emotional expression. To identify informational posts and to further ensure that only emotionally expressive content remained, we applied a pretrained BERT (Bidirectional Encoder Representations From Transformers)–based sentiment analysis model, which classified each initial post as positive, negative, or neutral. Only threads whose initial posts were labeled as positive or negative were kept, while threads with neutral sentiment were removed because they contained little or no emotional content. After this screening and sentiment-based filtering, the final dataset for analysis comprises 23,633 threads initiated by 9137 patients.

### Emotion Extraction

We capture both the presence and strength of emotions expressed in the initial posts. We apply the BERT [56] model to extract the 8 emotions and explore their effects on 5 community response categories (detailed description in section Community Response Measurements). As it was impractical to annotate emotions in all 23,633 initial posts manually, we used a machine learning–based approach to extract emotions from the posts. For the 8 emotions, the score for each emotion ranges from 0 to 10, where 0 indicates that the emotion is not expressed in the post, and 10 indicates the highest level of emotional strength. The details of the extraction process are described in Multimedia Appendices 1 and 2, and the distribution of the estimated scores for each emotion is presented in Table 1.

**Table 1:** Distribution of the predicted emotion scores.

Emotion	Observations	Mean (SD)	Minimum	Maximum
Surprise	23,633	1.66 (0.46)	0.08	2.73
Anticipation	23,633	4.46 (0.86)	0.81	5.69
Joy	23,633	1.78 (1.31)	0.02	5.22
Sadness	23,633	3.28 (1.09)	0.02	4.86
Trust	23,633	3.37 (0.69)	1.83	5.68
Disgust	23,633	2.52 (1.01)	0	3.84
Fear	23,633	3.76 (1.42)	0	5.23
Anger	23,633	1.6 (0.94)	0	3.19

### Community Response Measurements

In this study, the unit of analysis is per thread, and we examine community members’ responses to the initial post within each thread in 5 categories and summarize them in Table 2.

**Table 2:** Definition and explanation of community response categories.

Community response category	Definition	Explanation
Responder count	The number of distinct participants, aside from the initial poster, contributing to the thread within 7 days.	A higher number of responders can make the original poster feel that more community members care about them and are interested in the topic. Additionally, increased participants generally demonstrate that the community is active and welcoming.
Reply volume	The number of replies to a thread within 7 days, excluding the original poster’s self-replies.	This metric provides the original posters with a sense of the level of interest and attention generated by their initial post. A higher reply volume indicates greater engagement and interest in the topic.
Average reply length	The mean word count in replies, excluding self-replies by the initial poster, over the established time intervals (eg, 7 days).	This metric offers an estimate of the depth and quality of engagement from participants. Longer replies may indicate that participants are deeply engaging with the topic.
Response promptness	The time at which a thread receives a designated number of replies, excluding the initial poster’s self-replies. Specifically, it measures the time in seconds taken (in logarithm) for a thread to reach the first 10 replies.	Response promptness reflects how quickly the thread obtains initial responses. For the original posters, a quicker response can indicate immediate interest or urgency regarding their posts. This can be particularly important for time-sensitive topics or when the poster is seeking rapid feedback from the community.
Response relevance	The average topical relevance between the original post and all replies received within 7 days.	This measure evaluates whether community replies meaningfully address the concerns or information expressed in the original post. To compute this score, Sentence-BERT^a^ is used to generate embeddings for the original post and for all replies within a fixed time window (eg, 7 days). Semantic similarity (eg, cosine similarity, range from –1 to 1) is calculated for each reply-post pair. The response relevance is then computed as the average similarity of all replies falling within that time period. Higher scores indicate that replies are strongly aligned with the poster’s topic, while lower scores suggest off-topic, generic, or mismatched responses.

^a^BERT: Bidirectional Encoder Representations From Transformers.

### Empirical Analysis

We use the following ordinary least squares regression model (equation 1) to evaluate the impact of expressed emotion intensity in an initial post on subsequent community responses. The independent variables are the emotion-intensity scores for the 8 primary emotions. The model additionally controls for the medical context described in the post, user attributes, prior user participation behavior, and a range of linguistic and content-based features of the post. The dependent variables, independent variables, and all control variables are summarized in Table 3.

CommunityResponses = α_0_ + α_1_PostEmotions + α_2_PostHealth + α_3_AuthorHealthHistory + α_4_AuthorCommunityActivity + α_5_PostChar + *ε* (1)

**Table 3:** Summarization of the dependent variable, independent variable, and control variables.

Variables			Description
**Dependent variable**			
	CommunityResponses	In our analysis, we treat each of the 5 categories of community response measures as a dependent variable individually. Thus, we conduct 5 separate analyses on our data: 1 for responder count, 1 for reply volume, 1 for average reply length, 1 for response promptness, and 1 for response relevance.	
**Independent variable**			
	PostEmotions	We take the independent variable in the form of scores of the 8 emotion dimensions expressed in the initial post, for example, surprise, anticipation, joy, sadness, trust, disgust, fear, and anger.	
**Control variables**			
	PostHealth	PostHealth is a set of features regarding the number of diseases, diagnoses, drugs, examinations, procedures, and symptoms mentioned in the initial post. We used an LLM^a^, gpt-3.5-turbo-0125 [57], to identify and count those terms. Multimedia Appendix 3 provides more details about the identification process. These features can indicate the complexity of the patients’ conditions expressed in the initial post, which may be related to how other community members respond to the initial post and lead to more targeted, in-depth discussions.	
	AuthorHealthHistory	AuthorHealthHistory is a group of features containing the initial poster’s medical histories, including the number of diagnoses, surgeries, chemotherapy, radiation, target-therapy, and hormonal-therapy the initial poster had before starting this thread. This information is retrieved from the system-generated signature entered by the member. These features indicate the complexity of the patients’ overall health conditions, which may affect how community members respond to the author, fostering more specific and detailed discussions.	
	AuthorCommunityActivity	AuthorCommunityActivity represents the community participation behaviors of the initial post’s author, including the number of threads the author has initialized before starting the focal one, and the number of replies the author has written to others (excluding self-replies) before initializing this focal post. The reason for including this control variable is that prior research suggests that user behavior in social communities can be influenced by others’ actions and history [43,58,59]. Community members who have seen the author’s previous contributions may respond differently based on prior interaction and established reputation within the community.	
	PostChar	PostChar represents additional characteristics of the initial post, including the number of images in the post, whether the initial poster has signatures, the day of the week the post is created, and other linguistic features, which are extracted using LIWC^b^ [60]. Examples of LIWC features include word count of the post, number of “I,” “we,” and “you” in the post, and number of family, friend, body, money, and death-related words. These features represent the initial post’s content and visibility, which further impact how community members interpret and interact with the initial post.	

^a^LLC: large language model.

^b^LIWC: linguistic inquiry and word count.

## Results

The results of the analysis are presented in Table 4 regarding the effects of the independent variable (ie, the 8 primary emotions) on subsequent community responses across the 5 categories. Multimedia Appendix 4 reports the comprehensive results for other variables included in equation 1. The numbers in the parentheses indicate the SEs. In Table 4, columns 1 and 2 report the impacts of emotions on the responder count and the number of replies, respectively, which show significant similarities across the 2 categories. Emotions such as surprise (*P*<.001 for responder count and *P*=.03 for number of replies) and anticipation (*P*<.001 for both responder count and number of replies) negatively influence the responder count and reply volume, indicating that higher levels (greater intensity) of these emotions in the initial post significantly decrease the number of participants and the number of replies in the thread. Conversely, emotions such as joy (*P*<.001 for both responder count and number of replies), sadness (*P*=.003 for responder count and *P*=.02 for reply volume), trust (*P*<.001 for responder count and *P*=.047 for reply volume), fear (*P*<.001 for responder count and *P*=.02 for reply volume), and anger (*P*<.001 for both responder count and reply volume) positively affect the number of responders and replies, with increases in emotional intensity associated with more active participation. Disgust has no significant impact on the responder count but significantly increases the reply volume. Among all emotions, sadness has the most substantial effect, with each unit increase in sadness intensity attracting an average of 1.68 (SD 0.57) additional users to respond with 2.19 (SD 0.87) more replies in the thread. Column 3 demonstrates that the stronger expressions of anticipation (*P*<.001) and anger (*P*<.001) lead to longer replies, whereas higher-intensity presence of joy (*P*<.001) and sadness (*P*=.02) in the initial post tends to result in shorter replies. Anger and sadness exert the strongest but opposite effects on the average reply length. Column 4 shows that surprise (*P*<.001), anticipation (*P*<.001), and trust (*P*<.001) positively affect response promptness, meaning that greater emotional intensity in these categories delays the time it takes for an initial post to receive a certain number of replies (in this case, 10 replies). In contrast, posts expressing a higher level of intensity in joy (*P*<.001), fear (*P*<.001), and anger (*P*<.001) prompt quicker responses from other community members. Column 5 indicates that, when the original post expresses higher-intensity anticipation (*P*<.001) and fear (*P*=.007), community members are more likely to respond in a way that remains topically consistent with the post. However, greater intensity of joy (*P*<.001) is related to lower topical relevance, which suggests that replies tend to diverge from the original post’s content.

**Table 4:** Empirical results of emotions (independent variables only).^a^

Independent variables		Responder count (1)	Reply volume (2)	Average reply length (3)	Response promptness (4)	Response relevance (5)
**Surprise**						
	Coefficient value	–0.70^a^	–0.61^b^	1.60	0.15^a^	0.01
	SE	0.18	0.27	1.25	0.04	0.003
**Anticipation**						
	Coefficient value	–1.06^a^	–1.04^a^	4.67^a^	0.21^a^	0.03^a^
	SE	0.12	0.18	0.84	0.03	0.002
**Joy**						
	Coefficient value	0.94^a^	1.16^a^	–3.73^a^	–0.17^a^	–0.01^a^
	SE	0.10	0.16	0.73	0.02	0.002
**Sadness**						
	Coefficient value	1.68^c^	2.19^b^	–9.30^b^	–0.00	–0.02
	SE	0.57	0.87	4.04	0.13	0.01
**Trust**						
	Coefficient value	0.75^a^	0.62^b^	–2.47	0.17^a^	–0.003
	SE	0.19	0.29	1.32	0.04	0.003
**Disgust**						
	Coefficient value	0.17	0.54^b^	0.45	–0.06	0.002
	SE	0.17	0.26	1.18	0.04	0.003
**Fear**						
	Coefficient value	0.66^a^	0.40^c^	1.25	–0.10^a^	0.01^c^
	SE	0.10	0.15	0.69	0.02	0.002
**Anger**						
	Coefficient value	0.59^a^	0.81^c^	9.17^a^	–0.15^a^	–0.003
	SE	0.14	0.22	0.99	0.03	0.003

^a^*P*<.001.

^b^*P*<.05.

^c^*P*<.01.

Table 5 summarizes the control variables.

**Table 5:** Control variables.

Variables	Source
PostHealth	See Multimedia Appendix 4
AuthorHealthHistory	See Multimedia Appendix 4
AuthorCommmunityActivity	See Multimedia Appendix 4
PostChar	See Multimedia Appendix 4

To ensure the validity of our findings, we expanded our analysis to include various time windows (1, 2, 3, 10, and 30 days) and to compare the effects of different emotions. The results illustrate that, across all time intervals, sadness predominantly affects responder count, reply volume, and average reply length. Higher-intensity expressions of anger also have a strong impact on average reply length, while increases in anticipation intensity exert the most substantial influence on response promptness and response relevance among all emotions. Joy remains a relatively influential emotion across all 5 response categories. The detailed analysis process is demonstrated in Multimedia Appendix 5.

## Discussion

Our findings show that both the type and intensity of emotions expressed in patients’ initial posts are associated with different levels of community engagement and different response behaviors in OCCs, and that opposite emotions elicit similar rather than opposing responses, which confirms our hypothesis that the emotions shaping community engagement in OCCs extend beyond a simple positive-negative polarization. Specifically regarding RQ1, higher intensity of joy, sadness, trust, fear, and anger increase responder participation and the volume of reply, while surprise and anticipation decrease them. Sadness has the largest overall effect. Stronger expressions of anticipation and anger lead to longer replies, whereas higher-intensity joy and sadness are associated with shorter replies. Surprise, anticipation, and trust slow response speed, whereas joy, fear, and anger accelerate it. In addition, higher-intensity anticipation and fear increase topical relevance in replies, while joy reduces it. Furthermore, with respect to RQ2, this study also shows that for most opposing emotion pairs (eg, surprise vs anticipation, joy vs sadness, and fear vs anger), their effects on engagement move in parallel rather than in opposing directions. These results indicate that emotional expressions convey situational appraisals (social information) and simultaneously serve as social signals that affect how community members allocate attention, effort, and timing when providing support.

Across the 5 response dimensions, our results show that different emotional expressions influence multiple dimensions of community engagement in distinct ways, for example, whether members respond, when they respond, how much effort they invest, and how relevant their responses are. Certain emotions, including joy, sadness, anticipation, and anger, exert particularly strong effects across responder count, reply volume, reply length, response speed, and topical relevance, although the direction and magnitude of these effects vary by dimension. These findings support RQ1 by demonstrating that emotional expressions do not affect engagement uniformly; instead, different types of emotions guide attention, effort, timing, and content alignment in qualitatively different ways. This pattern is consistent with the EASI framework, which emphasizes that emotions function as social information and social signals that guide observers’ interpretations and behavioral decisions.

At the same time, our results indicate that emotional intensity amplifies signaling strength but does not override situational meaning. As emotional expressions are measured as continuous intensity scores, our analysis captures how increasing emotional strength alters engagement behaviors. Higher intensity generally increases the likelihood and scale of community response, but these effects are conditional on emotion type and response dimension. Emotional intensity, therefore, enhances the importance and clarity of emotional signals, which makes situational appraisals more visible to observers, without changing the situational context conveyed by a given emotion. This finding extends prior work that treats emotions categorically or dichotomously by showing that intensity complements, rather than replaces, the informational role of emotion type.

Regarding RQ2, our findings reveal that emotions defined as opposites in Plutchik’s framework do not consistently function as opposing social signals and elicit opposite engagement behaviors in OCCs. Instead, for most opposing emotion pairs (eg, surprise vs anticipation, joy vs sadness, and anger vs fear), the impacts on the 5 types of community responses move in parallel rather than opposing directions. A straightforward interpretation might be that opposite emotions receive similar reactions or community engagement because they are expressed with comparable intensity. However, our descriptive statistics do not support this explanation. As shown in our descriptive statistics, opposite emotions differ substantially in both their prevalence and intensity distributions. For example, sadness is expressed more frequently and with higher average intensity than its opposite emotion, joy; likewise, fear and anger also show distinct levels of occurrence and magnitude. As these opposite emotions do not share similar intensity patterns, the parallel effects observed in our models cannot be attributed to intensity alone. Instead, our results suggest that community members respond primarily to the situational context, such as facing uncertainty, confronting threats, or processing important outcomes, conveyed by emotional expressions rather than to emotional valence. For instance, both surprise and anticipation are about patients’ reactions to unpredictable situations; both joy and sadness are related to patients’ important milestones or accomplished events; both fear and anger are linked to potential threats to patients [50]. When emotions share similar appraisal meanings, they can elicit similar patterns of engagement even when they are theoretically defined as opposites. In addition, not all opposing emotion pairs behave identically; for example, 2 opposing pairs of emotions, namely surprise vs anticipation, and joy vs sadness, demonstrate completely opposite impacts across all community response categories. For example, initial posts expressing surprise or anticipation tend to receive a delayed and reduced number of replies from fewer responders. In contrast, posts expressing joy or sadness prompt quicker and more numerous replies. These contrasting response patterns may be caused by the situations described in the initial posts, for example, an unpredictable scenario vs a fact. Specifically, when other community members encounter descriptions of unpredictable situations, which are associated with surprise and anticipation, they may need more time to come up with thoughtful responses. In contrast, accomplished facts, which cause gains or losses to patients and further evoke joy or sadness, tend to elicit more immediate and numerous replies as the community members may be clearer about how to react, for example, celebrating successes or providing comfort for difficult issues. These patterns further support the EASI framework by showing that emotional expressions guide observers’ interpretations of situational context and appropriate response strategies.

Overall, this study contributes to health care, psychological, and sociological research by clarifying how emotions serve as social information and social signals in high-stress health contexts. First, this study extends the EASI model by showing that emotions defined as opposites do not necessarily function as opposing social signals in OCCs. Opposite emotions can elicit similar engagement behaviors not because they share comparable intensity, but because they convey similar situational context, such as uncertainty, threat, or outcome significance, from the perspective of community members. This finding refines EASI by demonstrating that observers’ responses are driven primarily by inferred situational context rather than emotional valence alone. In this sense, emotional expressions help community members infer underlying circumstances and determine appropriate forms of engagement, consistent with prior work showing that post content influences interaction patterns in online health communities [20,61,62]. Second, by modeling emotions as continuous intensity scales, this study provides a more precise account of how emotional communication affects support dynamics in OCCs. By integrating Plutchik’s emotion framework with the EASI perspective, we show that these effects are caused by the combination of emotion type and emotional intensity, offering insights beyond what polarity-based sentiment analysis has discovered. Emotional intensity clearly matters, but it is not independent of emotion type or appraisal meaning; instead, it amplifies the signaling strength of emotions and influences participation, effort, timing, and topical relevance in community responses. Third, our findings confirm the key tenets of the EASI model by demonstrating that different emotions expressed in patients’ initial posts are associated with distinct patterns of community engagement and response. When different emotions are expressed, community members react differently in terms of whether they respond, how quickly they respond, how much effort they invest, and how closely their replies align with patients’ concerns.

Our findings also have practical implications for patients, OCC platforms, and health care practitioners by clarifying how emotional expressions influence the distribution, timing, and nature of peer support in digitally mediated health communities. From the patient’s perspective, these findings help set realistic expectations about how OCCs may react and what responses they may receive. Patients may benefit from understanding that variations in response volume, speed, or depth are often a natural outcome of community dynamics rather than a reflection of the importance, legitimacy, or severity of their situation. For example, posts expressing certain emotions (eg, joy, sadness, anger, or anticipation) may be more likely to receive rapid or numerous responses, whereas others may elicit fewer or delayed replies. Increased awareness of these patterns can reduce feelings of neglect, self-blame, or disappointment and help patients interpret their online experiences more constructively. Such awareness may also encourage patients to engage more confidently with peer support resources throughout their cancer journey.

From the OCC platform and community management perspective, the findings provide actionable guidance for improving the effectiveness and equity of social support. OCCs can train moderators to recognize posts that are less likely to attract spontaneous engagement and proactively highlight or promote these posts to the community. More importantly, early prediction of low-engagement posts based on the emotions expressed enables scalable neglect-mitigation strategies. Given the large volume and length of user-generated posts in OCCs, manual monitoring is often infeasible. Emotion-aware models that predict engagement early can help identify posts at risk of being overlooked and support timely intervention. Such systems allow moderators to deploy targeted strategies aligned with the emotions expressed in the posts, such as prioritizing reassurance-oriented responses for posts expressing anticipation, or providing follow-up explanations for posts expressing surprise. By intervening strategically and broadly, OCCs can improve participation, encourage more balanced information exchange, and strengthen inclusive and supportive community environments [5,63,64].

From the health care practitioner’s perspective, our findings can inform how clinicians guide patients in using online support resources as part of psychosocial support. By understanding the relationship between emotional expression and community response patterns, health care providers can better prepare patients for their online interactions, help them interpret engagement outcomes, and reduce anxiety associated with delayed or limited responses. Clinicians may incorporate such guidance into consultations to encourage appropriate and beneficial engagement with OCCs.

In addition, health care providers, OCC facilitators, and technical teams can collaborate to develop emotion-aware systems that automatically identify posts expressing heightened distress, uncertainty, or fear. These systems can support early detection and prioritization of patients who may benefit from timely psychosocial support, counseling, or patient navigation services. By complementing existing screening and triage processes, such approaches can enhance care coordination and improve the overall quality of patient support.

This study has limitations. One limitation of this study is that our data come from a single long-standing cancer community with its own communication norms, which limits the generalizability of our findings to other health conditions or support platforms. In addition, emotional expressions in online posts may not fully represent patients’ true emotional states. Individuals often moderate, soften, or mask their emotions when communicating in a public forum, which means that the emotional intensity captured in their written posts may underestimate or misrepresent their actual feelings. As our analysis relies on expressed emotion as the independent variable, this moderation can weaken the correspondence between true emotional experience and textual emotional signals, which may potentially weaken the observed relationships between emotional expression and community responses. Furthermore, different community members may interpret the same emotional expression in varied ways based on their personal experiences, coping styles, or communication preferences, which introduces natural variability in how emotions are understood and reacted to within the community.

Future research can improve these limitations in several ways. First, expanding the analysis to other health conditions, support platforms, or culturally diverse communities would help assess the generalizability of these emotional dynamics and reveal whether different patient populations exhibit distinct emotional communication patterns. Second, studies may incorporate complementary qualitative or mixed method approaches to better understand how patients choose to express or withhold emotions in OCCs, and how these choices affect the interpretation and impact of their posts. Third, future work could examine how emotions evolve throughout the entire conversation rather than focusing solely on the initial post, offering deeper insight into the dynamics of emotional exchange and support over time. Fourth, researchers might explore how individual differences influence both emotional expression and community responses, such as for patients with different illness stages, coping strategies, or social support needs. Finally, investigating how emotions and other forms of content (eg, informational requests, narratives, or disclosures) interact to affect peer support could broaden our understanding of the mechanisms underlying engagement in online health communities and inform the design of more responsive and patient-centered support systems.

## Data Availability

The data used in this study are publicly accessible through Breastcancer.org.

## Appendix

Multimedia Appendix 1 Emotion extraction process.

Multimedia Appendix 2 Instructions to annotators.

Multimedia Appendix 3 Medical-term extraction.

Multimedia Appendix 4 Empirical results.

Multimedia Appendix 5 Process for robustness check.

## Abbreviations

BERT: Bidirectional Encoder Representations From Transformers
EASI: emotions as social information
OCC: online cancer community
