# Supplementary material for: Emotion Expression in Breast Cancer Support Seeking: Empirical Study of an Online Community
Source: JMIR Med Inform. 2026 Apr 13;14:e83674. doi: 10.2196/83674 (PMC13122135; doi:10.2196/83674)
Supplement: Multimedia Appendix 1 [file medinform_v14i1e83674_app1.docx]

Our emotion extraction process involves three steps: creating a training set by manual annotations, developing a machine learning model, and evaluating the model performance.

**Section S1. Emotion Annotation**

We randomly selected 2,500 initial posts from the dataset to create a gold standard for training the model. Two research assistants, who were familiar with OCCs, annotated the initial posts independently. To ensure the accuracy of the annotations, the annotators received detailed coding instructions (e.g., definitions of each emotion, examples of indicators, and illustrations) and training, including exercises and tests. Full instructions are in Multimedia Appendix 2. For each initial post, the annotator read the post and assigned a score from 0 to 10 for each of the eight emotions, where a score of 0 indicates that the emotion is not expressed in the post, and 10 indicates the highest level of emotional strength. As noted in our limitations, different individuals may interpret the same emotional expression differently based on their personal experiences, coping styles, and communication preferences, introducing natural variability in how emotions are perceived. The same applies to the annotation process, as each annotator may form a different impression of the emotions expressed in a post. To illustrate this variability, Table S1 reports the average score for each emotion assigned by each annotator, the mean score across both annotators, and the mean absolute difference between them. The results show that Annotator B consistently assigns higher emotion intensity scores than Annotator A.

**Table S1.** Annotation results.

| Emotion | Observations | Mean (Annotator A) | Mean (Annotator B) | Mean (Both Annotators) | Mean Absolute Difference |
| --- | --- | --- | --- | --- | --- |
| *Surprise* | 2,500 | 0.63 | 2.23 | 1.43 | 2.27 |
| *Anticipation* | 2,500 | 2.46 | 5.37 | 3.92 | 3.88 |
| *Joy* | 2,500 | 2.85 | 3.01 | 2.93 | 3.12 |
| *Sadness* | 2,500 | 1.32 | 3.12 | 2.22 | 2.9 |
| *Trust* | 2,500 | 1.97 | 5.96 | 3.97 | 4.43 |
| *Disgust* | 2,500 | 0.91 | 2.28 | 1.59 | 2.39 |
| *Fear* | 2,500 | 1.65 | 2.51 | 2.08 | 2.74 |
| *Anger* | 2,500 | 0.88 | 1.08 | 0.98 | 1.59 |

The average of the scores from the two annotators was used as the final score for an emotion. As a result, each initial post received eight scores corresponding to the eight emotions. We present the distribution of the emotion scores for the 2,500 annotated initial posts in Table S2.

**Table S2.** Distribution of the annotated emotion scores.

| Emotion | Observations | Mean | Standard Deviation | Minimum | Maximum |
| --- | --- | --- | --- | --- | --- |
| *Surprise* | 2,500 | 1.43 | 1.28 | 0 | 8.50 |
| *Anticipation* | 2,500 | 3.92 | 2.02 | 0 | 9.00 |
| *Joy* | 2,500 | 2.93 | 2.51 | 0 | 10.00 |
| *Sadness* | 2,500 | 2.22 | 1.91 | 0 | 9.50 |
| *Trust* | 2,500 | 3.97 | 1.65 | 0 | 9.50 |
| *Disgust* | 2,500 | 1.59 | 1.69 | 0 | 9.00 |
| *Fear* | 2,500 | 2.08 | 2.24 | 0 | 10.00 |
| *Anger* | 2,500 | 0.98 | 1.54 | 0 | 10.00 |

**Section S2. BERT Model Development**

The 2,500 annotated posts were used to train a machine learning model for automated emotion extraction from the remaining posts in the dataset. Machine learning, including natural language processing (NLP), methods are widely used to analyze user-generated contents in Online Health Communities [1-6]. In particular, the Bidirectional Encoder Representations from Transformers (BERT) [7] model has shown outstanding performance in various NLP tasks (e.g., document classification [8], named entity recognition [9], etc.) since it was pre-trained on diverse text corpora and can be easily fine-tuned for a specific task. In this study we used a BERT-base model to extract emotions from the initial posts and assign a score for each emotion for each post.

Typically, when a fine-tuned BERT is used for classification, the model minimizes the cross-entropy loss between the true categorical labels and predicted probabilities over the categories for the training set. However, given that emotion scores in this study are numerical, we modified the last layer in BERT to predict a continuous score in the range of [0, 10]. Like in regression analysis, we adopted the mean-squared-error (MSE) loss rather than the cross-entropy loss function typically used in categorical label prediction. The MSE is calculated using Equation (S1):

MSE =$\frac{\sum_{i=0}^{n} \left( y_{i}-p_{i} \right)^{2}}{n}$ (S1)

Where $y_{i}$ is the true emotion score, $p_{i}$ is the predicted emotion score, $i$ is the index of the post, $n$ is the total number of posts.

We fine-tuned a separate model for exacting each emotion. A 70-10-20 split is used: 70% of the posts were used for training, 10% for validation and 20% for testing. Since processing very long posts can be computationally expensive, the maximum number of tokens was set to 512. This means the model only processed the first 512 tokens in each post. This limit is sufficient to capture the full content of nearly all posts in our dataset, because 512 tokens correspond to approximately 384 words, while posts in our dataset average 161 words in length, and only 1423 out of 23643 (6%) posts exceed 384 words.

Following prior studies using BERT [7, 10-12] and our own experimentations, we set the key hyperparameters as follows: number of epochs = 3; batch size = 32; learning rate = 0.00001. We used the Huggingface [13] implementation of fine-tuned BERT.

**Section S3. Model Evaluation**

Table S3 presents the average MSE values for the eight emotions for the test set. As MSE is used as the loss function, low MSE values are preferred. The results show that *Surprise* and *Anger* have the lowest MSEs with the values of 1.45 and 1.50, respectively. *Joy* and *Fear* received the highest MSE values with 3.70 and 3.18 respectively.

**Table S3.** Performance of the Emotion Detection Model (Ordered with Increasing MSE).

| Emotion | Mean-squared-error |
| --- | --- |
| *Surprise* | 1.45 |
| *Anger* | 1.50 |
| *Trust* | 1.75 |
| *Disgust* | 2.10 |
| *Anticipation* | 2.57 |
| *Sadness* | 2.63 |
| *Fear* | 3.18 |
| *Joy* | 3.70 |

**Section S4. Robustness Check**

To further assess the validity of our BERT-based emotion extraction, we conducted a robustness evaluation using ChatGPT, with the results summarized in Table S4. We employed ChatGPT-3.5-Turbo in two configurations: zero-shot and fine-tuned. In the zero-shot setting, the model assigned emotion intensity without task-specific training, which reply on its pretrained knowledge only. In contrast, the fine-tuned version was additionally trained on our annotated data for better emotion intensity estimation. Using the same evaluation procedure described in Section S3, we compared the MSE across the eight primary emotions. As shown in Table S4, the BERT model achieves lower MSE than both ChatGPT configurations for most emotions, which represents a more accurate intensity estimation. In addition to its stronger performance, BERT can be used without additional costs, which provides a reliable and cost-effective solution for large-scale emotion analysis in this study.

**Table S4.** Robustness evaluation of emotion intensity extraction models.

|  | **Mean-Squared-Error** | | |
| --- | --- | --- | --- |
| **Emotion** | BERT | GPT-3.5 Turbo Zero-shot | GPT-3.5 Turbo finetuned |
| *Surprise* | 1.45 | 5.74 | 1.73 |
| *Anger* | 1.50 | 4.34 | 2.07 |
| *Trust* | 1.75 | 8.30 | 2.71 |
| *Disgust* | 2.10 | 4.09 | 1.85 |
| *Anticipation* | 2.57 | 7.51 | 4.06 |
| *Sadness* | 2.63 | 5.63 | 2.40 |
| *Fear* | 3.18 | 5.58 | 3.47 |
| *Joy* | 3.70 | 9.33 | 5.59 |

## Reference

1. Jones J, Pradhan M, Hosseini M, et al. Novel Approach to Cluster Patient-Generated Data Into Actionable Topics: Case Study of a Web-Based Breast Cancer Forum. JMIR Med Inform. 2018;6:e45. doi: 10.2196/medinform.9162

2. Shah AM, Lee KY, Hidayat A, et al. A text analytics approach for mining public discussions in online cancer forum: Analysis of multi-intent lung cancer treatment dataset. Int J Med Inform. 2024;184:105375. doi: 10.1016/J.IJMEDINF.2024.105375

3. Dreyfus B, Chaudhary A, Bhardwaj P, et al. Application of natural language processing techniques to identify off-label drug usage from various online health communities. Journal of the American Medical Informatics Association. 2021;28:2147–54. doi: 10.1093/JAMIA/OCAB124

4. Jelodar H, Wang Y, Orji R, et al. Deep Sentiment Classification and Topic Discovery on Novel Coronavirus or COVID-19 Online Discussions: NLP Using LSTM Recurrent Neural Network Approach. IEEE J Biomed Health Inform. 2020;24:2733–42. doi: 10.1109/JBHI.2020.3001216

5. Rodrigues RG, das Dores RM, Camilo-Junior CG, et al. SentiHealth-Cancer: A sentiment analysis tool to help detecting mood of patients in online social networks. Int J Med Inform. 2016;85:80–95. doi: 10.1016/J.IJMEDINF.2015.09.007

6. Yin Z, Sulieman LM, Malin BA. A systematic literature review of machine learning in online personal health data. Journal of the American Medical Informatics Association. 2019;26:561–76. doi: 10.1093/JAMIA/OCZ009

7. Devlin J, Chang M-W, Lee K, et al. BERT: Pre-training of Deep Bidirectional Transformers for Language Understanding. NAACL HLT 2019 - 2019 Conference of the North American Chapter of the Association for Computational Linguistics: Human Language Technologies - Proceedings of the Conference. 2018;1:4171–86.

8. Turchin A, Masharsky S, Zitnik M. Comparison of BERT implementations for natural language processing of narrative medical documents. Inform Med Unlocked. 2023;36:101139. doi: 10.1016/J.IMU.2022.101139

9. Babaian T, Xu J. Entity recognition from colloquial text. Decis Support Syst. 2024;179:114172. doi: 10.1016/J.DSS.2024.114172

10. Romanowski B, Ben Abacha A, Fan Y. Extracting social determinants of health from clinical note text with classification and sequence-to-sequence approaches. Journal of the American Medical Informatics Association. 2023;30:1448–55. doi: 10.1093/JAMIA/OCAD071

11. Tan RSYC, Lin Q, Low GH, et al. Inferring cancer disease response from radiology reports using large language models with data augmentation and prompting. Journal of the American Medical Informatics Association. 2023;30:1657–64. doi: 10.1093/JAMIA/OCAD133

12. Zhou H, Austin R, Lu SC, et al. Complementary and Integrative Health Information in the literature: its lexicon and named entity recognition. Journal of the American Medical Informatics Association. 2024;31:426–34. doi: 10.1093/JAMIA/OCAD216

13. Wolf T, Debut L, Sanh V, et al. HuggingFace’s Transformers: State-of-the-Art Natural Language Processing. EMNLP 2020 - Conference on Empirical Methods in Natural Language Processing, Proceedings of Systems Demonstrations. 2020;38–45. doi: 10.18653/V1/2020.EMNLP-DEMOS.6
