# Supplementary material for: Emotion Expression in Breast Cancer Support Seeking: Empirical Study of an Online Community
Source: JMIR Med Inform. 2026 Apr 13;14:e83674. doi: 10.2196/83674 (PMC13122135; doi:10.2196/83674)
Supplement: Multimedia Appendix 2 [file medinform_v14i1e83674_app2.docx]

Thank you for participating in the data annotation job. This job requires you to assign emotion scores to online user-generated messages (including threads and replies). All messages come from https://community.breastcancer.org/.

The emotion score assignment is based on Plutchik's wheel of emotions and we focus on eight emotions: joy, sadness, anger, fear, anticipation, disgust, trust, and surprise. The following descriptions may help you better understand each emotion:

1. **Joy**

- **Similar words**: *Excited, Pleased*
- **Typical sensations**: *Sense of energy and possibility*
- **What is Joy telling you?** *Life is going well*
- **How can Joy help you?** *Sparks creativity, connection, gives energy*

1. **Sadness**

- **Similar words:** Bummed, Loss
- **Typical sensations**: Heavy
- **What is Sadness telling you?** Love is going away
- **How can Sadness help you?** Focus on what's important to us

1. **Anger**

- **Similar words**: Mad, Fierce
- **Typical sensations**: Strong and heated
- **What is Anger telling you?** Something is in the way
- **How can Anger help you?** Energize to break through a barrier

1. **Fear**

- **Similar words**: Stressed, Scared
- **Typical sensations**: Agitated
- **What is Fear telling you?** Something I care about is at risk
- **How can Fear help you?**Protect what we care about

1. **Anticipation**

- **Similar words**: Curious, Considering
- **Typical sensations**: Alert and exploring
- **What is Anticipation telling you**? Change is happening
- **How can Anticipation help you?** Look ahead, look at what might be coming

1. **Disgust**

- **Similar words**: Distrust, Rejecting
- **Typical sensations**: Bitter & unwanted
- **What is Disgust telling you?** Wrong; rules are violated
- **How can Disgust help you?** Notice something unsafe or wrong

1. **Trust**

- **Similar words**: Accepting, Safe
- **Typical sensations**: Warm
- **What is Trust telling you?** This is safe
- **How can Trust help you?** Be open, connect, build alliance

1. **Surprise**

- **Similar words**: Shocked, Unexpected
- **Typical sensations**: Heart pounding
- **What is Surprise telling you?** Something new happened
- **How can Surprise help you?** Pay attention to what's right here

During the annotation process, you should read through the message and assign scores (0-10) to each of the eight emotions, with 0 indicating no expression of the emotion and 10 indicating an extremely high level of the emotion expressed in the message. The format of the annotation is presented below. You’ll given excel sheets and each row in the sheet is one annotation task. You’ll need to finish 2,500 tasks by the end of this job.

| Content | joy | sadness | anger | fear | anticipation | disgust | trust | surprise |
| --- | --- | --- | --- | --- | --- | --- | --- | --- |
| Example text1 | 3 | 3 | 0 | 3 | 8 | 0 | 5 | 2 |
| Example text2 | 9 | 0 | 0 | 3 | 1 | 1 | 2 | 10 |
