# Supplementary material for: Emotion Expression in Breast Cancer Support Seeking: Empirical Study of an Online Community
Source: JMIR Med Inform. 2026 Apr 13;14:e83674. doi: 10.2196/83674 (PMC13122135; doi:10.2196/83674)
Supplement: Multimedia Appendix 3 [file medinform_v14i1e83674_app3.docx]

We use the GPT3.5 Turbo model to extract medical terms from posts and self-replies. GPT3.5 is a large language model (LLM) which was trained on massive amounts of text. Therefore, it is able to perform extremely well on various kinds of Natural Language Processing tasks even without the need of labeled data. The medical terms we extract include drug names, treatments, exams, procedures, symptoms, diagnosis and disease names. We use a zero-shot approach where no labels are provided. Below is the prompt that we used to extract medical terms from each post and self-reply:

Prompt: *“Identify medical terms in this post and categorize them into these categories: drugs, treatments, exams, procedures, symptoms, diagnosis, and disease names. Provide the answer in the following format: drugs: drug1name, drug2name etc, treatments: treatment1, treatment2 etc... I want the output to be something which can easily be parsed in python. That is, do not add anything else to your response. Here is the post [the post].”*

We validated the output by manually ensuring the correctness of the extracted medical terms for a sample of posts. We listed 10 sample posts and their medical term extraction results here.

**Post 1**

*I just got a phone call from my mom. Bad news. My aunt was dx today. She is elderly and had a stroke a few years back that left her with dementia (sp?)...doctor recommends not treating the cancer and just making sure she is not in pain. This is the pits. I kind of have to agree. She has an extremely short memory. Everytime she went in for treatment would be like finding out for the first time that you had bc. My aunt is the sweetest women in the world. She loves company even if she doesn't always remember who you are. I have seen her when she gets scared. I wouldn't want to see her go through that everytime she looked down wondering where her breast went or why they were poking more needles in her. I wish i was closer. I just want to hug her. This is really hard on my mom and dad. She lived with them for a few years after her stroke. I know this is tearing them up. Sorry for rambling here. It just aches..........*

**Medical Terms Identified:**

- Drugs: None
- Treatments: Cancer treatment (unspecified)
- Exams: None
- Procedures: None
- Symptoms: Pain; Memory loss; Aches
- Diagnosis: Cancer (dx)
- Disease_names: Stroke; Dementia; Cancer; Breast cancer

**Post 2**

*Am 1 week post-dissection (7 NEG nodes...couldn't find the sentinel due to prev. exc. biopsy...my luck!) and was going to have my acrylic nail “fill” but now I am wondering...maybe no more acrylics? Menopause killed my nails and all they did was split and break and get hangnails so I'm not sure which might contribute more to an infection...real, crummy nails or fake "healthy" ones. And who the heck would I ask except for all you experts here! I don't have LE but am wondering if you ladies still get your nails done and if so, do you do any acrylics? Forewarned is forearmed! Thanks!*

**Medical Terms Identified:**

- Drugs: None
- Treatments: None
- Exams: None
- Procedures: Lymph node dissection; Sentinel biopsy (attempted); Excisional biopsy
- Symptoms: Nail splitting; Hangnails
- Diagnosis: None
- Disease_names: Menopause; Infection; Lymphedema

**Post 3**

*I am not certain where to post this....I have finished chemo and my year of herceptin......I had stage 2A breast cancer bilateral mas, 35 rads, 4 a/c, 4 taxotere/herceptin then a year of herceptin.....I stopped menstruating during the first chemos....but during herceptin started again....I am 41......my Oncologists wants us to use Lupron over 6 months to put me in early menopause even though I did not have Er/PR receptors. Anyone else gone through this????*

**Medical Terms Identified:**

- Drugs: Herceptin; Taxotere; Lupron
- Treatments: Chemotherapy; Radiation (35 rads); Hormonal suppression
- Exams: None
- Procedures: Bilateral mastectomy
- Symptoms: Amenorrhea
- Diagnosis: Stage 2A breast cancer; ER/PR negative
- Disease_names: Breast cancer; Early menopause

**Post 4**

*It really seems to help to read these posts and realize that there are so many of us. My youngest daughter is due to have her first baby July 15. I have 2 other daughters, and the tradition has become that I sew a super blanket for each grandchild. Well, I got hit with the bc diagnosis right in the middle of that project, and now I've nearly finished her blanket. So many emotions crowd through my head..depressing ones and uplifting ones too. To be a woman....what a huge job. So, I am not thinking about the 15th either...thanks for the suggestion. I'm wishing you good thoughts and happiness. Keep the faith...baby*

**Medical Terms Identified:**

- Drugs: None
- Treatments: None
- Exams: None
- Procedures: None
- Symptoms: None
- Diagnosis: Breast cancer
- Disease_names: Breast cancer

**Post 5**

*Hi Friends, I am trying to understand something here. My therapist, when I was having treatment, said this to me. Most people with LE type "pockets" of swelling and mine is a gradual swelling in the forearm. Which do you have please? thanks so much LE hugs jinny*

**Medical Terms Identified:**

- Drugs: None
- Treatments: Lymphedema therapy
- Exams: None
- Procedures: None
- Symptoms: Swelling
- Diagnosis: Lymphedema
- Disease_names: Lymphedema

**Post 6**

*I will be starting ACT in a few weeks after I complete another surgery. I would like to stay on disability for chemo -- does anyone have experience with this being approved? thanks!*

**Medical Terms Identified:**

- Drugs: None
- Treatments: ACT chemotherapy; Surgery
- Exams: None
- Procedures: Surgery
- Symptoms: None
- Diagnosis: None
- Disease_names: None

**Post 7**

*My Onc has referred me to a lymphedema clinic but I'm on a waiting list - number 20 - to get an appointment. Does anyone have any suggestions as to what I can do while I wait to get an appointment? I have LE in my left breast area and around my side under my arm. It's getting painful under my arm. Any suggestions would be appreciated. CrazyAunt*

**Medical Terms Identified:**

- Drugs: None
- Treatments: Lymphedema clinic referral
- Exams: None
- Procedures: None
- Symptoms: Pain; Swelling
- Diagnosis: Lymphedema
- Disease_names: Lymphedema

**Post 8**

*I am just diagnosed of bc and about having my mastectomy on 4.7.07. Right now I'm very scared and don't know what is out there waiting for me. Doc has mentioned about chemotherapy. Can those who have gone through these share their experience? I'm 43, have 2 young kids. I will do all I could to survive. But rightnow I really don't know what to do. Some told me must take care of my food, get t-shirt or organic food. Can anybody give some ideas?*

**Medical Terms Identified:**

- Drugs: None
- Treatments: Chemotherapy
- Exams: None
- Procedures: Mastectomy
- Symptoms: Fear; Anxiety
- Diagnosis: Breast cancer
- Disease_names: Breast cancer

**Post 9**

*Morning Ladies...Up again at 3am and have been quietly playing solitaire for ages seems like. I've read that sleep will be better after A/C is over, and I'm taking Trazedone to help with sleep! Still have two more to go before the taxal, was supposed to have #3 on Friday but had a tooth absess - big one, and doc wouldn't do chemo. So went right away to Dentist - who got me in asap on his afternoon without patients - good guy, and now on antibiotics. Will probably do #3 on this Friday. Was pretty diappointed, got all psyched up to not cry - actually did better this poke, and even took one of my Emends....and now will probably have to be short for this Friday's tx because insurance will have a spazz because it's not time for me to get more. Am thankful for insurance but this is a dumb rule. Anyway....just had to ramble this morning. Board looks kind of slow, hope everyone is feeling OK and not too down after the holidays. Good luck on your surgery joyous, listen to your body and rest. Feel my hug. We can endure it and be trained by it. suzy*

**Medical Terms Identified:**

- Drugs: Trazedone; Antibiotics; Emend
- Treatments: AC chemotherapy; Taxol
- Exams: None
- Procedures: None
- Symptoms: Insomnia; Tooth abscess pain
- Diagnosis: Tooth abscess
- Disease_names: Tooth abscess

**Post 10**

*I am a Stage II Grade 3 triple neg, with negative needle biopsy of single suspisious node (suspicious by palpation and US). Tumor near nipple and 2.6 cm; large hematoma happened during core biopsies, and MRI biopsies of calcifications in multiple dimetric disease class.... all this complicates the staging and treatment plans. I had 12 taxols and am 1/2 way through 4 FAC. Have not seen the PS yet, but possible I can have some type of TRAM. If needle recon is questionable, or other things are found at time of Sx, I could end up needing radiation (not good for recon). It's a catch-22, miss the opportunity for immediate recastion, and bear risk of needing radiation post-op. radiation changes the environment for good plastic surgery. I'm rather having rad radiation on top of their autologous adrect recon!!! This is extremely weary process. I'm so confused.*

**Medical Terms Identified:**

- Drugs: Taxol; FAC regimen
- Treatments: Chemotherapy; Radiation; TRAM reconstruction
- Exams: Needle biopsy; Core biopsy; MRI biopsy; Ultrasound
- Procedures: Biopsies; TRAM flap reconstruction; Surgery
- Symptoms: Hematoma
- Diagnosis: Stage II Grade 3 triple-negative breast cancer; Multicentric disease
- Disease_names: Breast cancer; Triple-negative breast cancer; Hematoma
