# Supplementary material for: Emotion Expression in Breast Cancer Support Seeking: Empirical Study of an Online Community
Source: JMIR Med Inform. 2026 Apr 13;14:e83674. doi: 10.2196/83674 (PMC13122135; doi:10.2196/83674)
Supplement: Multimedia Appendix 4 [file medinform_v14i1e83674_app4.docx]

**Table S1.** Empirical results of emotion scores.

|  | |  | (1) | (2) | (3) | (4) | (5) |
| --- | --- | --- | --- | --- | --- | --- | --- |
|  | |  | Responder Count | Reply  Volume | Average Reply Length | Response Promptness | Response Relevance |
| **Independent Variables** | | | | | | | |
|  | | Surprise | -0.70*** | -0.61* | 1.60 | 0.15** | 0.01 |
|  | |  | (0.18) | (0.27) | (1.25) | (0.04) | (0.003) |
|  | | Anticipation | -1.06*** | -1.04*** | 4.67*** | 0.21*** | 0.03*** |
|  | |  | (0.12) | (0.18) | (0.84) | (0.03) | (0.002) |
|  | | Joy | 0.94*** | 1.16*** | -3.73*** | -0.17*** | -0.01*** |
|  | |  | (0.10) | (0.16) | (0.73) | (0.02) | (0.002) |
|  | | Sadness | 1.68** | 2.19* | -9.30* | -0.00 | -0.02 |
|  | |  | (0.57) | (0.87) | (4.04) | (0.13) | (0.01) |
|  | | Trust | 0.75*** | 0.62* | -2.47 | 0.17*** | -0.003 |
|  | |  | (0.19) | (0.29) | (1.32) | (0.04) | (0.003) |
|  | | Disgust | 0.17 | 0.54* | 0.45 | -0.06 | 0.002 |
|  | |  | (0.17) | (0.26) | (1.18) | (0.04) | (0.003) |
|  | | Fear | 0.66*** | 0.40** | 1.25 | -0.10*** | 0.01** |
|  | |  | (0.10) | (0.15) | (0.69) | (0.02) | (0.002) |
|  | | Anger | 0.59*** | 0.81** | 9.17*** | -0.15*** | -0.003 |
|  | |  | (0.14) | (0.22) | (0.99) | (0.03) | (0.003) |
| **Health Related Features in Mentioned Initial Post** | | | | | | | |
|  | | Number of Diseases | 0.19** | 0.28** | 0.40 | -0.02 | -0.001 |
|  | |  | (0.06) | (0.09) | (0.43) | (0.01) | (0.001) |
|  | | Number of Diagnosis in Post | 0.16** | 0.13 | 0.09 | -0.04** | -0.001 |
|  | |  | (0.05) | (0.08) | (0.35) | (0.01) | (0.001) |
|  | | Number of Drugs | 0.07 | 0.07 | -1.25*** | -0.01 | -0.001 |
|  | |  | (0.04) | (0.06) | (0.27) | (0.01) | (0.001) |
|  | | Number of Exams | 0.07 | -0.01 | 0.08 | -0.02 | -0.003** |
|  | |  | (0.06) | (0.09) | (0.42) | (0.01) | (0.001) |
|  | | Number of Procedures | -0.14** | -0.06 | 3.12*** | 0.05** | 0.004*** |
|  | |  | (0.05) | (0.08) | (0.35) | (0.01) | (0.001) |
|  | | Number of Symptoms | -0.09** | -0.11* | -0.05 | 0.03*** | -0.000 |
|  | |  | (0.03) | (0.04) | (0.20) | (0.01) | (0.001) |
| **Author Health History (Before Focal Post)** | | | | | | | |
|  | | Number of Diagnosis | 0.06 | -0.10 | -0.36 | -0.02 | 0.003 |
|  | |  | (0.14) | (0.21) | (0.97) | (0.03) | (0.003) |
|  | | Number of Surgeries | -0.92*** | -1.06*** | -0.96 | 0.17*** | -0.001 |
|  | |  | (0.10) | (0.16) | (0.74) | (0.02) | (0.002) |
|  | | Number of Chemotherapies | 0.58*** | 0.63** | -1.65 | -0.06 | -0.01** |
|  | |  | (0.13) | (0.20) | (0.93) | (0.03) | (0.002) |
|  | | Number of Radiations | 0.05 | -0.06 | -4.85** | 0.03 | -0.01 |
|  | |  | (0.21) | (0.32) | (1.50) | (0.05) | (0.004) |
|  | | Number of Targeted Therapies | 0.72** | 0.77* | 1.51 | 0.02 | -0.002 |
|  | |  | (0.23) | (0.36) | (1.65) | (0.05) | (0.004) |
|  | | Number of Hormonal Therapies | 0.66*** | 0.53* | -1.18 | -0.12** | -0.001 |
|  | |  | (0.16) | (0.24) | (1.11) | (0.04) | (0.003) |
| **Author Community Activity (Before Focal Post)** | | | | | | | |
|  | | Number of Threads | 0.00 | 0.00 | -0.01** | -0.00* | -0.000 |
|  | |  | (0.00) | (0.00) | (0.00) | (0.00) | (0.000) |
|  | | Number of Replies | 0.00*** | 0.00*** | -0.00*** | -0.00*** | -0.000*** |
|  | |  | (0.00) | (0.00) | (0.00) | (0.00) | (0.000) |
| **Post Characteristics** | | | | | | | |
|  | | Number of Images | 0.27* | 0.41* | -0.29 | -0.04 | 0.000 |
|  | |  | (0.13) | (0.19) | (0.90) | (0.03) | (0.002) |
|  | | System Signature (Binary) | 0.62** | 1.00** | -1.21 | -0.16*** | -0.01 |
|  | |  | (0.17) | (0.26) | (1.22) | (0.04) | (0.003) |
|  | | User Generated Signature (Binary) | 0.79*** | 0.91*** | -3.23*** | -0.10*** | -0.01*** |
|  | |  | (0.11) | (0.18) | (0.81) | (0.03) | (0.002) |
|  | | Word Count | -0.00*** | -0.00*** | 0.05*** | 0.00*** | -0.000 |
|  | |  | (0.00) | (0.00) | (0.00) | (0.00) | (0.000) |
|  | | Monday | 0.00 | 0.00 | 0.00 | 0.00 | 0.000 |
|  | |  | (.) | (.) | (.) | (.) | (.) |
|  | | Tuesday | 0.21 | 0.41 | -0.23 | 0.01 | -0.004 |
|  | |  | (0.19) | (0.30) | (1.38) | (0.04) | (0.004) |
|  | | Wednesday | -0.02 | -0.16 | -0.35 | 0.04 | -0.002 |
|  | |  | (0.19) | (0.30) | (1.38) | (0.04) | (0.004) |
|  | | Thursday | -0.13 | -0.31 | 1.02 | 0.07 | -0.003 |
|  | |  | (0.20) | (0.30) | (1.39) | (0.05) | (0.004) |
|  | | Friday | -0.05 | -0.06 | 0.72 | 0.15** | -0.005 |
|  | |  | (0.20) | (0.30) | (1.40) | (0.05) | (0.004) |
|  | | Saturday | -0.49* | -0.56 | 4.93** | 0.28*** | 0.003 |
|  | |  | (0.21) | (0.32) | (1.47) | (0.05) | (0.004) |
|  | | Sunday | -0.38 | -0.24 | 3.97** | 0.16** | 0.002 |
|  | |  | (0.21) | (0.32) | (1.46) | (0.05) | (0.004) |
|  | Analytic | | -0.00 | -0.01 | -0.10 | 0.00 | -0.001*** |
|  |  | | (0.01) | (0.02) | (0.08) | (0.00) | (0.000) |
|  | Clout | | -0.01 | 0.00 | -0.03 | -0.00 | -0.000 |
|  |  | | (0.01) | (0.01) | (0.07) | (0.00) | (0.000) |
|  | Authentic | | 0.01 | 0.01 | 0.11** | 0.00 | 0.000 |
|  |  | | (0.01) | (0.01) | (0.04) | (0.00) | (0.000) |
|  | WPS | | -0.03*** | -0.03** | -0.08* | 0.00 | -0.000 |
|  |  | | (0.01) | (0.01) | (0.04) | (0.00) | (0.000) |
|  | Sixltr | | -0.09*** | -0.10*** | 0.42*** | 0.02*** | 0.000 |
|  |  | | (0.01) | (0.02) | (0.10) | (0.00) | (0.000) |
|  | Dic | | 0.06** | 0.05 | 0.54** | -0.03*** | 0.000 |
|  |  | | (0.02) | (0.03) | (0.15) | (0.00) | (0.000) |
|  | function | | 0.02 | 0.06 | -0.20 | 0.01 | -0.001 |
|  |  | | (0.04) | (0.06) | (0.26) | (0.01) | (0.001) |
|  | pronoun | | -0.03 | -0.38 | 4.24 | -0.09 | 0.01 |
|  |  | | (0.45) | (0.69) | (3.18) | (0.11) | (0.01) |
|  | ppron | | 22.46* | 19.18 | -106.94 | -7.80** | -0.24 |
|  |  | | (10.18) | (15.59) | (71.96) | (2.35) | (0.19) |
|  | i | | -22.51* | -18.91 | 102.33 | 7.88** | 0.23 |
|  |  | | (10.17) | (15.59) | (71.92) | (2.35) | (0.19) |
|  | we | | -22.08* | -18.14 | 102.32 | 7.88** | 0.23 |
|  |  | | (10.17) | (15.58) | (71.91) | (2.35) | (0.19) |
|  | you | | -22.55* | -18.97 | 103.38 | 7.91** | 0.23 |
|  |  | | (10.17) | (15.59) | (71.92) | (2.35) | (0.19) |
|  | shehe | | -21.99* | -18.41 | 102.30 | 7.85** | 0.23 |
|  |  | | (10.17) | (15.59) | (71.92) | (2.35) | (0.19) |
|  | they | | -22.49* | -18.90 | 101.97 | 7.92** | 0.23 |
|  |  | | (10.17) | (15.59) | (71.92) | (2.35) | (0.19) |
|  | ipron | | -0.16 | 0.15 | -4.94 | 0.11 | -0.01 |
|  |  | | (0.45) | (0.69) | (3.19) | (0.11) | (0.01) |
|  | article | | -0.10 | -0.10 | 0.47 | 0.01 | 0.01*** |
|  |  | | (0.05) | (0.08) | (0.36) | (0.01) | (0.001) |
|  | prep | | -0.05 | -0.09 | 0.47 | 0.00 | 0.01*** |
|  |  | | (0.05) | (0.07) | (0.33) | (0.01) | (0.001) |
|  | auxverb | | 0.02 | -0.03 | -0.23 | 0.02 | -0.001 |
|  |  | | (0.05) | (0.07) | (0.32) | (0.01) | (0.001) |
|  | adverb | | 0.03 | -0.01 | -0.04 | 0.00 | -0.001 |
|  |  | | (0.04) | (0.06) | (0.26) | (0.01) | (0.001) |
|  | conj | | -0.13** | -0.19** | 0.20 | 0.02* | 0.001 |
|  |  | | (0.04) | (0.06) | (0.27) | (0.01) | (0.001) |
|  | verb | | -0.08** | -0.04 | 0.36 | 0.02** | 0.002** |
|  |  | | (0.03) | (0.04) | (0.20) | (0.01) | (0.001) |
|  | adj | | 0.01 | 0.02 | 0.00 | 0.00 | 0.001 |
|  |  | | (0.03) | (0.05) | (0.22) | (0.01) | (0.001) |
|  | compare | | 0.01 | -0.01 | 0.13 | 0.03** | -0.001 |
|  |  | | (0.04) | (0.07) | (0.31) | (0.01) | (0.001) |
|  | interrog | | 0.02 | 0.07 | 2.14*** | -0.01 | -0.000 |
|  |  | | (0.05) | (0.07) | (0.34) | (0.01) | (0.001) |
|  | number | | -0.01 | -0.04 | 0.91*** | -0.00 | 0.003*** |
|  |  | | (0.03) | (0.05) | (0.21) | (0.01) | (0.001) |
|  | quant | | -0.05 | -0.07 | 0.18 | 0.02 | 0.002* |
|  |  | | (0.04) | (0.06) | (0.29) | (0.01) | (0.001) |
|  | social | | 0.07 | 0.08 | 0.53 | -0.00 | 0.001 |
|  |  | | (0.04) | (0.06) | (0.29) | (0.01) | (0.001) |
|  | family | | 0.10 | -0.04 | 0.51 | -0.03 | -0.000 |
|  |  | | (0.10) | (0.15) | (0.69) | (0.02) | (0.002) |
|  | friend | | 0.50** | 0.54** | -1.94* | -0.06* | 0.001 |
|  |  | | (0.13) | (0.20) | (0.91) | (0.03) | (0.002) |
|  | female | | -0.26** | -0.28* | -0.12 | 0.04 | 0.004* |
|  |  | | (0.09) | (0.14) | (0.63) | (0.02) | (0.002) |
|  | male | | -0.36** | -0.43** | -0.39 | 0.05* | 0.001 |
|  |  | | (0.10) | (0.15) | (0.69) | (0.02) | (0.002) |
|  | cogproc | | 0.04 | 0.11 | 0.26 | -0.01 | -0.000 |
|  |  | | (0.04) | (0.07) | (0.32) | (0.01) | (0.001) |
|  | insight | | 0.02 | -0.02 | -0.18 | -0.01 | 0.000 |
|  |  | | (0.05) | (0.08) | (0.39) | (0.01) | (0.001) |
|  | cause | | -0.03 | -0.09 | -0.57 | 0.01 | -0.001 |
|  |  | | (0.06) | (0.09) | (0.41) | (0.01) | (0.001) |
|  | discrep | | -0.11* | -0.17* | 0.21 | 0.03** | 0.002* |
|  |  | | (0.05) | (0.08) | (0.37) | (0.01) | (0.001) |
|  | tentat | | -0.13** | -0.18** | -0.33 | 0.04*** | -0.001 |
|  |  | | (0.04) | (0.06) | (0.28) | (0.01) | (0.001) |
|  | certain | | 0.11 | 0.12 | -0.04 | -0.01 | 0.000 |
|  |  | | (0.06) | (0.09) | (0.43) | (0.01) | (0.001) |
|  | differ | | -0.15** | -0.17 | -0.83* | 0.01 | -0.000 |
|  |  | | (0.06) | (0.09) | (0.39) | (0.01) | (0.001) |
|  | percept | | -0.21* | -0.28* | -1.64** | 0.08** | 0.002 |
|  |  | | (0.09) | (0.14) | (0.63) | (0.02) | (0.002) |
|  | see | | 0.40*** | 0.48** | -0.88 | -0.11*** | -0.007** |
|  |  | | (0.10) | (0.15) | (0.69) | (0.02) | (0.002) |
|  | hear | | 0.29* | 0.39* | 1.34 | -0.10** | -0.002 |
|  |  | | (0.11) | (0.17) | (0.80) | (0.03) | (0.002) |
|  | feel | | -0.05 | -0.03 | 1.13 | -0.03 | -0.001 |
|  |  | | (0.10) | (0.15) | (0.68) | (0.02) | (0.002) |
|  | bio | | -0.08** | -0.10* | -0.15 | 0.02** | 0.001 |
|  |  | | (0.03) | (0.04) | (0.21) | (0.01) | (0.001) |
|  | body | | 0.18*** | 0.15* | -2.07*** | -0.04** | -0.002* |
|  |  | | (0.04) | (0.07) | (0.31) | (0.01) | (0.001) |
|  | sexual | | -0.45*** | -0.45*** | 2.51*** | 0.10*** | 0.01*** |
|  |  | | (0.07) | (0.11) | (0.50) | (0.02) | (0.001) |
|  | ingest | | 0.05 | 0.17 | 0.47 | -0.02 | 0.002 |
|  |  | | (0.06) | (0.09) | (0.42) | (0.01) | (0.001) |
|  | drives | | 0.20** | 0.27* | 0.26 | -0.02 | -0.01** |
|  |  | | (0.07) | (0.11) | (0.51) | (0.02) | (0.001) |
|  | affiliation | | -0.31** | -0.41** | -0.10 | 0.04* | 0.003* |
|  |  | | (0.08) | (0.13) | (0.58) | (0.02) | (0.002) |
|  | achieve | | -0.02 | -0.06 | 0.44 | 0.01 | 0.002 |
|  |  | | (0.07) | (0.10) | (0.46) | (0.02) | (0.001) |
|  | power | | -0.16* | -0.22* | 0.04 | 0.01 | 0.004** |
|  |  | | (0.07) | (0.11) | (0.50) | (0.02) | (0.001) |
|  | reward | | -0.12 | -0.20 | -1.37** | 0.01 | 0.003* |
|  |  | | (0.07) | (0.11) | (0.52) | (0.02) | (0.001) |
|  | risk | | -0.27** | -0.37** | -0.36 | 0.05* | 0.01*** |
|  |  | | (0.09) | (0.14) | (0.63) | (0.02) | (0.002) |
|  | focuspast | | -0.06 | -0.18** | -1.14*** | 0.00 | -0.001* |
|  |  | | (0.03) | (0.05) | (0.21) | (0.01) | (0.001) |
|  | focuspresent | | 0.01 | -0.02 | -0.20 | -0.02** | -0.001** |
|  |  | | (0.03) | (0.04) | (0.19) | (0.01) | (0.000) |
|  | focusfuture | | 0.19*** | 0.19** | -1.01** | -0.03** | -0.002** |
|  |  | | (0.04) | (0.06) | (0.30) | (0.01) | (0.001) |
|  | relativ | | -0.07 | -0.00 | -1.07* | 0.03* | -0.004** |
|  |  | | (0.07) | (0.10) | (0.47) | (0.02) | (0.001) |
|  | motion | | 0.04 | -0.02 | 0.13 | -0.01 | 0.003* |
|  |  | | (0.07) | (0.10) | (0.46) | (0.02) | (0.001) |
|  | space | | -0.06 | -0.16 | -0.17 | -0.00 | 0.003** |
|  |  | | (0.06) | (0.09) | (0.44) | (0.01) | (0.001) |
|  | time | | 0.01 | -0.07 | -0.46 | -0.03* | 0.004** |
|  |  | | (0.06) | (0.09) | (0.42) | (0.01) | (0.001) |
|  | work | | -0.27*** | -0.33*** | -0.10 | 0.03** | -0.002* |
|  |  | | (0.04) | (0.06) | (0.29) | (0.01) | (0.001) |
|  | leisure | | -0.19** | -0.15 | -0.37 | 0.06*** | -0.000 |
|  |  | | (0.06) | (0.09) | (0.42) | (0.01) | (0.001) |
|  | home | | 0.74*** | 0.68*** | -0.95 | -0.13*** | -0.001 |
|  |  | | (0.10) | (0.16) | (0.73) | (0.02) | (0.002) |
|  | money | | -0.23** | -0.37** | -1.41** | 0.02 | 0.002 |
|  |  | | (0.07) | (0.11) | (0.49) | (0.02) | (0.001) |
|  | relig | | 0.56*** | 0.56*** | -3.37*** | 0.01 | 0.003 |
|  |  | | (0.09) | (0.14) | (0.66) | (0.02) | (0.002) |
|  | death | | 0.92*** | 1.20*** | 0.54 | -0.11** | -0.004 |
|  |  | | (0.16) | (0.24) | (1.10) | (0.04) | (0.003) |
|  | informal | | -0.18 | -0.21 | -2.87* | 0.05 | -0.002 |
|  |  | | (0.17) | (0.26) | (1.18) | (0.04) | (0.003) |
|  | swear | | 0.63** | 0.94** | 0.30 | -0.11** | -0.003 |
|  |  | | (0.19) | (0.29) | (1.33) | (0.04) | (0.003) |
|  | netspeak | | -0.14 | -0.25 | 3.04** | 0.01 | 0.003 |
|  |  | | (0.16) | (0.25) | (1.15) | (0.04) | (0.003) |
|  | assent | | 0.35 | 0.66* | 2.01 | -0.07 | 0.003 |
|  |  | | (0.20) | (0.30) | (1.39) | (0.05) | (0.004) |
|  | nonflu | | 0.44* | 0.51 | 0.67 | -0.06 | -0.002 |
|  |  | | (0.18) | (0.28) | (1.30) | (0.04) | (0.003) |
|  | filler | | 1.02** | 1.17* | 2.02 | -0.08 | -0.004 |
|  |  | | (0.31) | (0.47) | (2.18) | (0.07) | (0.01) |
|  | AllPunc | | 2.57 | -0.21 | 80.55 | 0.26 | -0.004 |
|  |  | | (6.44) | (9.86) | (45.50) | (1.49) | (0.12) |
|  | Period | | -2.59 | 0.20 | -80.46 | -0.27 | 0.004 |
|  |  | | (6.44) | (9.86) | (45.50) | (1.49) | (0.12) |
|  | Comma | | -2.59 | 0.20 | -79.82 | -0.26 | 0.004 |
|  |  | | (6.44) | (9.86) | (45.50) | (1.49) | (0.12) |
|  | Colon | | -2.47 | 0.42 | -80.75 | -0.31 | -0.001 |
|  |  | | (6.44) | (9.87) | (45.52) | (1.49) | (0.12) |
|  | SemiC | | -2.46 | 0.39 | -80.02 | -0.22 | 0.003 |
|  |  | | (6.44) | (9.87) | (45.55) | (1.49) | (0.12) |
|  | QMark | | -2.52 | 0.24 | -80.44 | -0.30 | 0.01 |
|  |  | | (6.44) | (9.86) | (45.50) | (1.49) | (0.12) |
|  | Exclam | | -2.51 | 0.25 | -81.14 | -0.27 | 0.004 |
|  |  | | (6.44) | (9.86) | (45.50) | (1.49) | (0.12) |
|  | Dash | | -2.63 | 0.16 | -80.03 | -0.26 | 0.01 |
|  |  | | (6.44) | (9.86) | (45.50) | (1.49) | (0.12) |
|  | Quote | | -2.52 | 0.48 | -80.48 | -0.28 | 0.003 |
|  |  | | (6.44) | (9.86) | (45.50) | (1.49) | (0.12) |
|  | Apostro | | -2.54 | 0.27 | -81.07 | -0.28 | 0.004 |
|  |  | | (6.44) | (9.86) | (45.50) | (1.49) | (0.12) |
|  | Parenth | | -2.66 | 0.13 | -79.84 | -0.25 | 0.01 |
|  |  | | (6.44) | (9.86) | (45.50) | (1.49) | (0.12) |
|  | OtherP | | -2.65 | 0.13 | -79.91 | -0.25 | 0.004 |
|  |  | | (6.44) | (9.86) | (45.51) | (1.49) | (0.12) |
|  | cons | | 5.61** | 8.64** | 36.59** | 6.11*** | 0.34*** |
|  |  | | (1.93) | (2.95) | (13.63) | (0.45) | (0.04) |

Notes: **P* < .05, ***P* < .01, ****P* < .001. Standard errors are in parentheses.
