# Supplementary material for: Emotion Expression in Breast Cancer Support Seeking: Empirical Study of an Online Community
Source: JMIR Med Inform. 2026 Apr 13;14:e83674. doi: 10.2196/83674 (PMC13122135; doi:10.2196/83674)
Supplement: Multimedia Appendix 5 [file medinform_v14i1e83674_app5.docx]

To ensure the validity of our findings, we expanded our analysis to include various time windows. We assessed the impacts of the eight emotions on responder count, reply volume, average reply length, and response relevance at varying time intervals (1 day, 2 days, 3 days, 10 days, and 30 days). Additionally, we evaluated the promptness of community responses by measuring how quickly a thread receives its first 1, 5, 15, and 20 replies. The additional analysis results show consistent impacts of the eight emotions on the five community response categories across different time frames (e.g., 1 day, 2 days, 3 days, 7 days, 10 days, and 30 days).

In addition, we plot all the results by generating a heatmap from normalized coefficients of all models to highlight the differential impacts of each emotion, as depicted in Figure 4. This comparative analysis examined four dependent variables across all time intervals (e.g., 1 day, 2 days, 3 days, 7 days, 10 days, and 30 days) and evaluated the promptness that threads receive their first 1, 5, 10, 15, and 20 replies.

In the heatmap, each row corresponds to the coefficient results from a regression analysis for a specific dependent variable, while each column represents an independent variable. The reason for the differing scales in regression results across dependent variables lies in their inherent scale differences. For instance, within the 7 days time interval, the coefficient of sadness to the average reply length is -9.2995, whereas its coefficient to the responder count, excluding the initial poster is 1.6814. Displaying true coefficient values directly in one single heatmap could overshadow smaller scale results due to the predominance of larger scale ones. To avoid this issue and ensure clarity in visualization, we normalize the coefficients for each regression (each row). In specific, we divide each coefficient by the largest absolute coefficient value found within its respective regression analysis. The normalization process is detailed further in Equations E1 and E2.

$$maxAbsoluteCoefficient_{d}=$$

$$\max\left( abs\left( TrueCoefficient_{dE} \right) \right|$$

$E\{Joy, Sadness, Anger, Fear, Anticipation, Surprise, Disgust, Trust\})$E1

$NomalizedCoefficient_{di}= \frac{TrueCoefficient_{di}}{maxAbsoluteCoefficient_{d}}$ E2

Where $d$ represents a dependent variable, and $TrueCoefficient_{dE}$ is the true coefficient of emotion $E$ in that regression with corresponding $d$, and $E\{Surprise, Anticipation, Joy, Sadness, Trust, Disguest, Fear, Anger\}$.


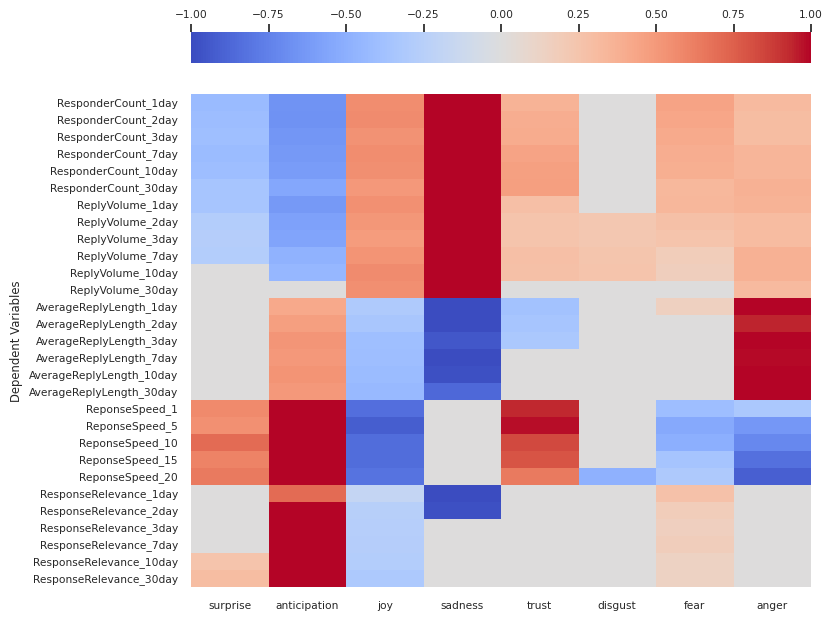


**Figure 4.** Comparison analysis results

Warm red hues indicate positive coefficients, signifying a positive effect on the dependent variables, whereas cool blue tones denote negative impacts. The intensity of the color correlates with the magnitude of coefficients, with darker shades representing stronger effects (larger coefficients) and lighter shades indicating milder influences (lower coefficients). Grey tone means no effect. The heatmap illustrates that, across all time intervals, sadness predominantly affects responder count, reply volume, and average reply length. Anger also has a strong impact on average reply length, while anticipation exerts the most substantial influence on response promptness and response relevance among all emotions. Joy is also a relative significant emotion across all five response categories.
